# Effects of Extreme Weather on Reproductive Success in a Temperate-Breeding Songbird

**DOI:** 10.1371/journal.pone.0080033

**Published:** 2013-11-05

**Authors:** Ivett Pipoly, Veronika Bókony, Gábor Seress, Krisztián Szabó, András Liker

**Affiliations:** 1 Department of Limnology, University of Pannonia, Veszprém, Hungary; 2 Department of Ecology, Szent István University, Budapest, Hungary; Columbia University, United States of America

## Abstract

The frequency of extreme meteorological events such as heat waves and rainstorms is predicted to increase with climate change. However, there is still little information about how extreme weather influences reproduction in animals. It may not only affect breeding success but might also alter offspring sex ratio if males and females are differentially sensitive to meteorological conditions during development. We investigated the relationship between meteorological conditions and reproductive success over 6 years in a house sparrow population in central Europe. We found that hatching success increased with the number of extremely hot days (daily maximum >31°C) and decreased with the number of extremely cold days (<16°C) during incubation, although the latter effect held only for clutches with relatively short incubation periods. Fledging success was unrelated to weather variables. However, the frequency of extremely hot days had a negative effect on fledglings’ body mass and tarsus length, although both of these traits were positively related to average temperature. Additionally, fledglings’ body mass increased with the length of period without rainfall before fledging. Male to female ratio among fledglings did not differ from 1:1 and did not vary with weather variables. The magnitude of the effects of extreme meteorological events was usually small, although in some cases comparable to those of ecologically relevant predictors of reproductive success. Our results indicate that meteorological conditions have complex effects on breeding success, as the effects of extreme weather can differ between different aspects of reproduction and also from the effects of overall meteorological conditions.

## Introduction

Global average temperature is increasing on the Earth, and this process has been getting faster in the last 50 years [1]. Besides climate warming, increases in the frequency and magnitude of meteorological extremities are also expected [2], as the observed and projected data predict more hot days, more extreme rainfalls and longer droughts for most regions of the Earth [3]. The largest anomalies are measured in summer when most biological productivity occurs, so this is probably the season when climate change will have its greatest impact on ecosystems [4].

 Wildlife species’ range, habitat, phenology, demographic and morphological traits can change in response to climate warming [5–8], and the magnitude of these responses depends on several ecological and life-history characteristics of the species [9]. Differential responses by different species (e.g. predator and prey) may lead to phenological mismatches which can alter the rates of reproduction and survival, causing decline in some populations and increase in others [10–12]. For example, Møller et al. [13] have found in a comparative study that birds that did not respond to recent climate change by shifting their spring migration phenology have declining breeding populations, whereas species that advanced their timing of migration have stable or increasing populations in Europe. This finding is supported by a similar study on the phenology of egg-laying [14]. Thus, climate change may have crucial fitness consequences in animal populations. Understanding and predicting these effects requires detailed knowledge about the effects of different aspects of weather on the biota. 

 Traditionally, meteorological conditions were included into the studies of reproductive success mostly as background variables [15], and the effects of weather *per se* on individuals or populations have been rarely studied up to recently [16–19]. Despite the recognized need for predicting the effects of increasing weather extremities [2,20], such effects are investigated mostly in connection with human health [3,21,22], whereas we know very little about the ability of animals to cope with such conditions [23]. Therefore, beyond the long-term phenological monitoring of populations [24,25], reproductive behaviour and fitness should be studied in relation to weather extremities to understand how meteorological events get translated into responses at the level of individuals and populations.

 In birds, prevailing weather can affect the main components of reproduction such as hatching success and fledging success in several ways. Low temperatures may make it difficult to maintain the optimal temperature of eggs (e.g. when parents have to interrupt incubation), and young nestlings that lack own thermoregulation are also very vulnerable to chilling [15]. Access to food may also be related to weather, either beacuse prey may be less available during certain meteorological circumstances [26], or because the ability of parents to collect food may be affected [27,28]. Besides the components of reproductive success, weather may also influence the sex ratio of offspring. One sex can be more sensitive to environmental conditions than the other [29,30], thus extreme or unfavorable weather may affect sons and daughters differently during their ontogeny; however, this phenomenon is yet little studied [31,32]. Furthermore, offspring sex ratio can also be altered by differential parental investment, e.g. parents in some species may benefit by producing more sons under favourable conditions (e.g. [33,34]). 

 In this study our goal was to understand the effects of prevailing weather and extreme meteorological events on the breeding biology of a hole-nesting sedentary bird species, the house sparrow (*Passer domesticus*). Specifically, we investigated how local temperature and precipitation during incubation and nestling development influence hatching success, body size and fledging success of nestlings, and brood sex ratio. We focused on two aspects of weather: the overall conditions during each period and the frequency of extremities.

## Methods

### Data collection

We studied the reproduction of house sparrows in a nest box-breeding population in the Kittenberger Zoological Garden of Veszprém, Hungary (N 47°05’32”, E 17°53’44”) from April to August each year between 2005 and 2010. Hungary has temperate climate affected by oceanic, continental and mediterranean climates; the study area is located in a moderately cool and moderately dry region. The most precipitation usually falls from May to July (58-71 mm per month) whereas the warmest period of a year is usually betweeen late July and early August. The monthly mean temperatures from April to July are between 10 °C and 25 °C [35], and the lowest and highest extremes at the study site were -2.2 °C  and 40.3 °C, respectively, during the study period.

Each nest box was checked at least twice a week, and the number of eggs or nestlings was recorded. Date of laying was either ascertained during laying, since house sparrows lay one egg per day [36], or estimated as 11 days minus hatching date if the clutch was found complete (average length of incubation period was 10.58 ± 0.08 (SE), n = 230 clutches). Date of hatching was either ascertained by checking the nest on consecutive days or estimated from the developmental state of nestlings when hatching had occurred in the inter-monitoring interval. Nestlings were ringed before fledging at the age of 10.2 ± 0.1 (SE) days, using an individual combination of one aluminium and three plastic rings, with two rings on each tarsus. Upon ringing, we measured each nestling’s body weight (± 0.1 g) by a spring balance and the length of the left tarsus (± 0.1 mm) by a vernier caliper. Brood size at 10 days of chick age is a good predictor of recruitment rate in house sparrows (Schwagmeyer and Mock 2008); since disturbing nests with older nestlings can cause premature fledging, we used the number of nestlings at the time of ringing (i.e. pre-fledging age) as proxy for the number of fledglings. There were 317 clutches where at least one nestling hatched; and 736 nestlings reached the pre-fledging age in 227 broods (Table S1). 

### Offspring sexing

Information about the sex of the offspring originated from two sources. On the one hand, sex was known from recapture and/or resighting data from the study area for n=92 individuals (51 males, 41 females) that hatched in the studied broods. On the other hand, the sex of 193 nestlings (89 males, 104 females) was determined by molecular genetic method. For this purpose, blood samples were taken upon ringing by brachial venipuncture, stored in Queen’s lysis buffer at room temperature until the laboratory procedures. Due to financial constraints we had to limit genetic analyses to 20 broods per year chosen randomly from all broods hatched between 2005-2007 (we only had blood samples from these years), summing up to 60 broods and 236 nestlings. We always sexed whole broods, i.e. each nestling being alive at the age of ringing in a given nest was sexed. Samples were analysed in 2011 in the molecular laboratory of the Department of Ecology, Institute of Biology, Faculty of Veterinary, Szent István University, Budapest. DNA was extracted using standard phenol-chloroform extraction [37]. Sex was determined by PCR amplification of the *CHD1-W* and *CHD1-Z* genes, using the 2550F/2718R primer pair [38]. This primer pair produced ambiguous results with some samples, so in these cases we repeated sexing using the P2 /P8 primers [39] to verify the results. PCR reactions (with both primer pairs) were performed using the conditions as described by the authors publishing the primers [38,39]. PCR products were evaluated by agarose gel-electrophoresis. To verify the molecular results, we additionally analysed the blood samples of n=39 individuals whose sex was known from resighting and/or recapture data. Sex determined by molecular analysis agreed with sex registered during resightings and/or recaptures in all but one case.

### Meteorological variables

Throughout the study, a meteorological station (HW WS 2350) about 2800 meters from the study area (N 47°10'16", E 17°93'14") collected data on daily minimum and maximum temperatures (°C) and daily amount of precipitation (mm). These data were used to create meteorological variables that characterize weather conditions for two periods for each nest: the incubation period of clutches (from the day of laying the penultimate egg to the day of first hatching), and the nestling period (from the day of first hatching to the day preceding the day of ringing and measuring). First we calculated two variables that represent the overall weather conditions during each period: daily mean temperature as the mean of daily minimum and maximum temperatures averaged over the period, and total amount of precipitation during the period. Then we calculated four variables to express the frequency or extent of extreme conditions during each period. 1) The number of hot days was defined as the number of days when daily maximum temperature was higher than 30.9°C, the 90th percentile of our daily maximum temperature data in April-August 2005-2010. This value corresponds well with the definition used in human meteorology, i.e. days with >30°C maximum temperature are considered heat days [35]. We also validated our definitions of extremities using a 100-years database measured in 1901-2000 ca. 100 km from our study site (Hungarian Meteorological Service, Budapest); the 90% percentile of daily maximum temperature in this dataset was 30.8°C, indicating that hot days by our definition were indeed rare extremities during the last century in our region. 2) The number of cold days was defined as the number of days when daily maximum temperature was below 15.9°C, the 10th percentile of our April-August data (15.3°C in the 100-years dataset). 3) The number of heavy rain days was defined as the number of days when the amount of daily precipitation was higher than 10 mm, the 90th percentile of our data (11.3 mm in the 100-year dataset; human meteorology also uses the 10 mm threshold) [35]. Finally, 4) the number of dry days was defined as the maximum number of consecutive days when no precipitation was recorded till the end of the incubaton or nestling period; this variable expresses the length of uninterrupted dry period preceding hatching or fledging, respectively. For example, if the last rainfall during the period occurred 5 days before the end of the period, then the number of dry days was 4, irrespective of the number of rainy days before the last rainy day. The length of continuous dry periods as defined above varied between 1-26 days, thus the entire incubation or chick rearing could coincide with a period without any rain. 

### Statistical analyses

We calculated the following variables to quantify components of reproductive success. Hatching success was the percentage of hatched eggs in those nests where at least one chick hatched. Fledging success was the percentage of hatched young that were alive at the age of ringing in those nests where at least one nestling reached that age. We excluded nests in which no chick hatched or no chick reached the age of ringing from the calculation of hatching and fledging success, respectively, because the period for which the meteorological variables should be calculated was not comparable with (i.e. was much shorter than) the incubation and nestling periods of successful nests. For each brood, we calculated the mean body mass and mean tarsus length of nestlings to avoid pseudo-replication because the values of siblings cannot be treated as non-independent data points. Sex ratio in each brood was expressed as the number of males divided by the total number of nestlings. Date was measured as the number of days from 1^st^ of January within each study year until the day of hatching of the first chick in each brood. Period length was defined as the length of incubation period in the analyses of hatching success, and as the length of nestling period in the analyses of fledging success (see above). Brood size was included in the analyses of nestlings' size as the number of nestlings in a brood at the age of ringing.

**Figure 1:**
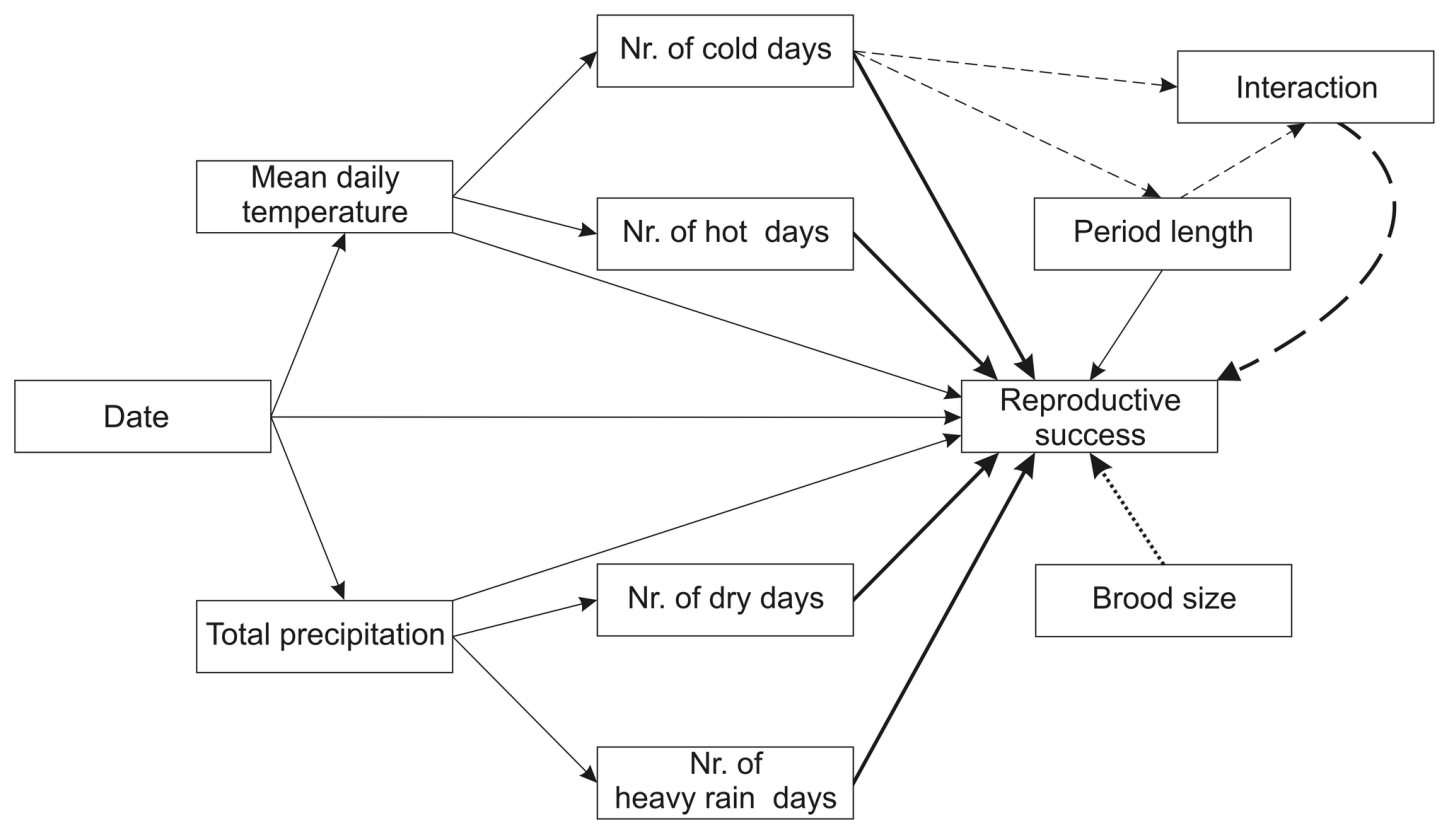
Model structure in SEM analyses. Thin lines stand for effects included in all models, thick lines for relationships that varied within model sets, dashed lines for paths contained only in the model set of hatching success, and the dotted line for the effect of brood size in model sets of nestlings’ body mass and tarsus length.

 We used structural equation modelling (SEM) to investigate the correlations between reproductive success and weather conditions. SEM is a multivariate statistical method particularly useful for decomposing the covariation within complex sets of multi-colinear variables [40,41]. We fitted structural equation models by the method of maximum likelihood using AMOS 20.0 [42]. Because the error distribution of our data was not normal, the 95% confidence intervals of path coefficients were estimated by bootstraping, with 9000 bootstrap samples for each model [41]. For each of the five measures of reproductive success (dependent variables), we constructed a set of nested *a priori* models (Tables S2, S3, S4, S5, S6). In each model set, the full model estimated reproductive success as function of both the two overall and four extreme meteorological variables (Figure 1). Further candidate models contained various plausible combinations of these six weather variables and a „null model” with no weather effects (Table S2, S3, S4, S5, S6). 

 All models of hatching success contained the effect of the number of cold days on period length, because cold weather can lengthen the incubation period [43,44]. This path was not included for the rest of the dependent variables (fledging success and nestlings' size), because period length was set by researchers at ca. 10 days in these cases by measuring the nestlings around 10 days of age. For hatching success, we constructed additional models including the interaction between the number of cold days and the length of incubation period (Table S2), because we expected that the effect of cold days may depend on whether or not parents adjust incubation length to the cold [45,46]. All models in each model set included the direct effect of date on reproductive success, since date may influence breeding not only via its impact on weather but also through other seasonal changes, such as the seasonal decline of food availability or offspring value and thereby parental effort [47,48]. As potential confounding variables, all models included the effect of period length (incubation period or nestling period) on reproductive success, and models of nestlings’ body mass and tarsus length also contained the effect of brood size (e.g. sibling competition).

 Each model set was evaluated using the information-theoretic approach, comparing the candidate models by their Akaike’s information criterion (AIC) value [49]. For each model we calculated its AIC difference from the „best model” (i.e. the model with the lowest AIC-value in the model set) and its Akaike weight which estimates the probability that the model is actually the best model in the model set. Then we used the model-averaging approach to calculate model-averaged parameter estimates and unconditional variances for each variable based on the whole model set [49]. All variables were z-transformed prior to the analyses, as recommended for SEM analyses [41], thereby the values of parameter estimates can be interpreted as standardized effect sizes, i.e. the amount of change in units of SD in the dependent variable’s value in response to 1 SD increase in the predictor’s value. According to Cohen's rule of thumb, effects above 0.2, 0.5 and 0.8 are considered small, medium and large, respectively [50], whereas mean effect size ranges 0.22 - 1.7 in ecological studies [51]. Thus, we defined important effects as paths with >|0.2| parameter estimates, and/or 95% confidence intervals >|0.2| at one side and not including zero (or including zero but very close to it) on the other side; note that confidence intervals including zero do not necessarily mean the lack of effect [49,52].

Additionally, we investigated whether the relationship between nestlings' body size and weather differed between male and female offspring by using multigroup analysis [41,42], which compares the variance–covariance matrices of SEM models between groups. We ran the full model shown in Figure 1 for both body mass and tarsus length in two ways: first constraining the parameter estimates of paths from meteorological variables towards the dependent variable to have the same value for both sexes, then allowing them to differ between sexes. The fit of these two models were compared by χ^2^ tests based on minimum discrepancy (Ĉ_min_) [42].

 Although weather or reproductive success may change non-linearly over the season, the quadratic effect of date was not included into our models because graphs indicated that the seasonal variation of both temperature and precipitation can be sufficiently described by linear models in our study period (Figure S1). Any potential quadratic effect of weather on reproductive success was modeled by the simultaneous inclusion of overall and extreme meteorological variables. Although consecutive broods in the same nest box (presumably by the same pair) are repeated measures, we did not include random effects into our models because the current implementations of SEM cannot handle random factors. To evaluate the importance of repeated measures, we built linear mixed-effect models for each dependent variable and compared pairs of models with and without the random effect (i.e. nestbox ID) using likelihood ratio tests in R [53]. We found that models without the random effect fit our data similarly well as models containing the random effect (∆AIC<2, p>0.156 in all cases). The non-independence of within-brood siblings’ data was handled by using their averages per brood (see above). 

### Ethics statement

This study of house sparrows, including capturing, measuring and blood sampling of the birds and monitoring their breeding, was in accordance with Hungarian laws and was approved by Balaton Upland National Park (permission number: 9135-2/2004, 2255/2008) and by the Middle Transdanubian Inspectorate for Environmental Protection, Natural Protection and Water Management (permission number: 31559/2011). The directory and the workers of the Kittenberger Zoological Garden of Veszprém kindly ensured the study area. No any other special premission was required for our work.

## Results

Overall, the effects of weather variables on reproductive success were small, as effect sizes ranged between 0 and 0.43 (absolute values; Table 1). Nevertheless, some confidence intervals included moderate or even strong effects of weather on hatching success and nestling morphology (Table 1). Notably, the range of weather effects were comparable in magnitude to those of other ecologically relevant predictors of breeding success, i.e. date, length of incubation period, nestling age and brood size (0.03-0.41; Table 1).

**Table 1:** Model-averaged parameter estimates [95% confidence intervals] for five measures of reproductive success as dependent variables.

Path in SEM			Hatching success	Fledging success	Body mass	Tarsus length	Sex ratio
**Daily mean temperature**	**→**	**Dependent variable**	**-0,06 [-0.27; 0.15]**	**0.01 [-0.15; 0.18]**	**0.43 [0.06; 0.81]**	**0.43 [0.07; 0.79]**	**-0.02 [-0.34; 0.30]**
**Total amount of precipitation**	→	**Dependent variable**	**-0.05 [-0.25; 0.14**]	**0.04 [-0.13; 0.21**]	**0.08 [-0.10; 0.26**]	**-0.01 [-0.16; 0.14**]	**0.00 [-0.28; 0.28**]
**Nr. of hot days**	→	**Dependent variable**	**0.14 [-0.03; 0.32**]	**0.03 [-0.06; 0.11**]	**-0.10 [-0.25; 0.05**]	**-0.11 [-0.25; 0.03**]	**-0.07 [-0.29; 0.14**]
**Nr. of cold days**	→	**Dependent variable**	**-0.20 [-0.51; 0.11**]	**-0.02 [-0.10; 0.06**]	**-0.01 [-0.09; 0.08**]	**0.04 [-0.06; 0.15**]	**0.02 [-0.12; 0.15**]
**Nr. of dry days**	→	**Dependent variable**	**0.01 [-0.06; 0.08**]	**0.03 [-0.05; 0.11**]	**0.23 [0.03; 0.42**]	**0.05 [-0.05; 0.15**]	**-0.07 [-0.27; 0.14**]
**Nr. of heavy rain days**	→	**Dependent variable**	**0.00 [-0.12; 0.13**]	**-0.04 [-0.15; 0.07**]	**-0.02 [-0.13; 0.09**]	**-0.02 [-0.11; 0.08**]	**-0.10 [-0.35; 0.15**]
**Nr. of cold days × incubation period**	→	**Dependent variable**	**0.24 [-0.05; 0.53**]	**-**	**-**	**-**	**-**
*Date*	→	*Dependent variable*	*-0.04 [-0.21; 0.14*]	*-0.13 [-0.37;0.10*]	*-0.30 [-0.63; 0.03*]	*0.04 [-0.15; 0.23*]	*-0.16 [-0.61; 0.29*]
*Incubation period/ Nestling period*	→	*Dependent variable*	*-0.20 [-0.46; 0.06*]	*-0.15 [-0.38; 0.09*]	*0.22 [-0.07; 0.51*]	*0.41 [0.03; 0.79*]	*-*
*Brood size*	→	*Dependent variable*	*-*	*-*	*0.03 [-0.11; 0.17*]	*0.18 [-0.07; 0.43*]	*-*
Date	→	Daily mean temperature	0.86 [0.27; 1.44]	0.85 [0.31; 1.38]	0.84 [0.74; 0.94]	0.84 [0.30; 1.38]	0.85 [-0.15; 1.85]
Date	→	Total amount of precipitation	0.20 [-0.09; 0.48]	0.05 [-0.11; 0.21]	0.05 [-0.09; 0.18]	0.05 [-0.11; 0.20]	0.01 [-0.25; 0.27]
Daily mean temperature	→	Nr. of hot days	0.64 [0.14; 1.15]	0.74 [0.24; 1.25]	0.75 [0.61; 0.89]	0.75 [0.24; 1.26]	0.82 [-0.17; 1.80]
Daily mean temperature	→	Nr. of cold days	-0.65 [-1.16; -0.14]	-0.43 [-0.82; -0.05]	-0.44 [-0.58; -0.29]	-0.44 [-0.83; -0.05]	-0.47 [-1.22; 0.28]
Total amount of precipitation	→	Nr. of dry days	-0.42 [-0.83; -0.01]	-0.39 [-0.75; -0.02]	-0.39 [-0.63; -0.16]	-0.39 [-0.76; -0.02]	-0.43 [-1.15; 0.28]
Total amount of precipitation	→	Nr. of heavy rain days	0.92 [0.31; 1.52]	0.92 [0.36; 1.48]	0.93 [0.83; 1.03]	0.93 [0.37; 1.49]	0.91 [-0.13; 1.95]
Nr. of cold days	→	Incubation period	0.36 [-0.02; 0.74]	­	-	-	-
Nr. of cold days	→	Interaction	0.96 [0.34; 1.58]	-	-	-	-
Incubation period	→	Interaction	0.09 [-0.10; 0.27]	-	-	-	-

A higher parameter value indicates higher effect size along the path in SEM. Paths highlighted in bold and italics show the effects of weather and non-weather variables on dependent variables, respectively.

**Figure 2:**
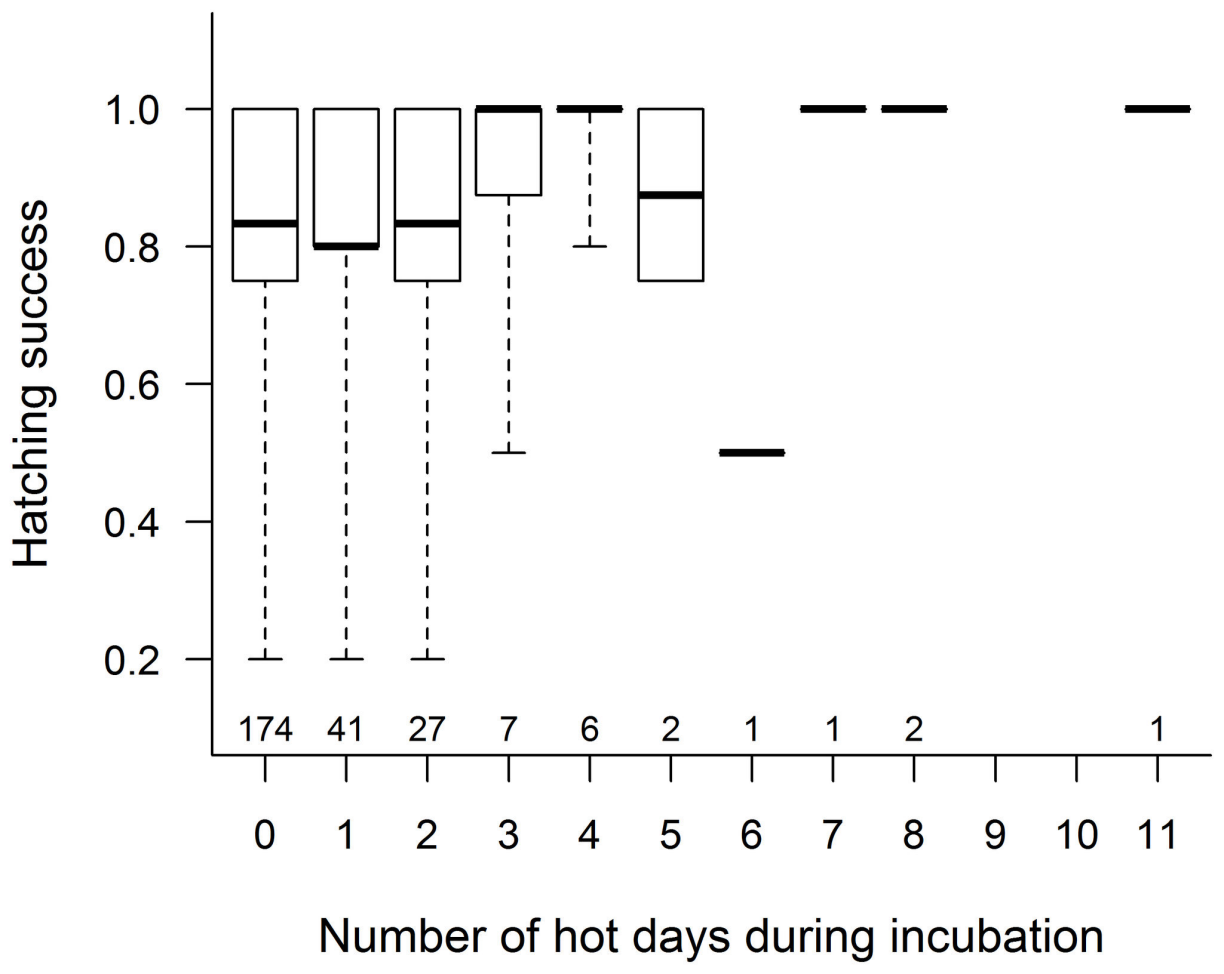
Relationship of hatching success with the number of hot days during incubation. Box plots show the median (thick line), interquartile range (box) and the range of data (whiskers); sample sizes are shown below each box.

**Figure 3:**
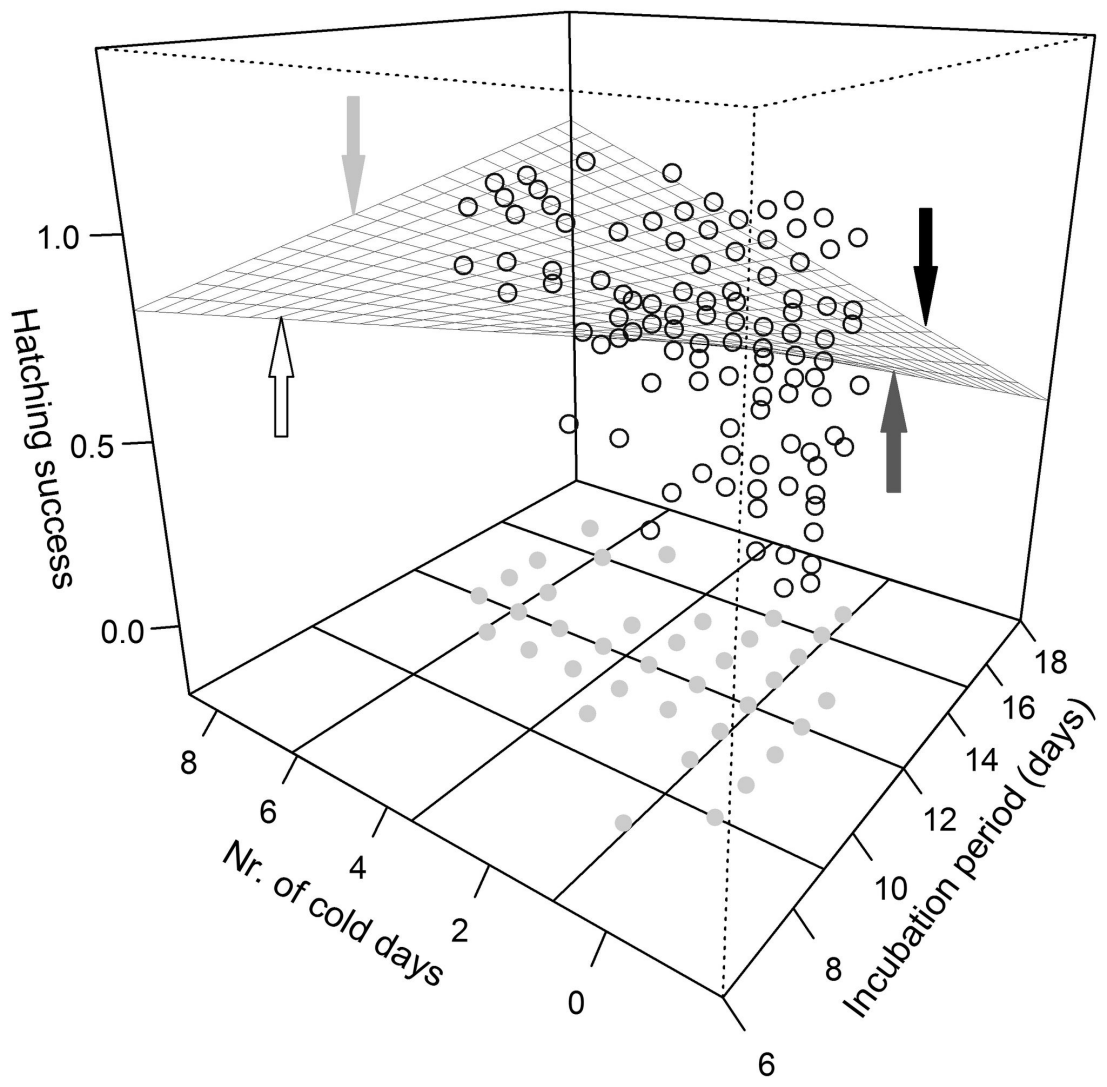
Relationship of hatching success with the number of cold days and length of incubation period. The warped regression plane was fitted from a linear regression to illustrate the interacting effects of the two predictors on hatching success. Open circles are the data points in 3D space defined by the three variables. Grey dots on the bottom grid of the graph show the relationship between the number of cold days and length of incubation period. Arrows highlight the slopes of the relationships between hatching success and number of cold days when incubation is short (white) or long (black), and between hatching success and length of incubation when number of cold days is high (light-grey) or low (dark-grey).

 For hatching success, two important meteorological effects emerged (Table 1, Table S2). A greater proportion of eggs hatched when there were more extremely hot days (Figure 2) and fewer extremely cold days during incubation. However, the latter effect held only for clutches with short incubation periods (Figure 3, see regression plane edge indicated by white arrow). More cold days were associated with increased incubation period length (Figure 3, bottom grid and grey dots), and longer incubation in cold periods was correlated with higher hatching success (Figure 3, light-grey arrow), but prolonged incubation during non-cold periods was associated with reduced hatching success (Figure 3, dark-grey arrow), leading to a positive relationship between the number of cold days and hatching success for long incubation periods (Figure 3, black arrow). For fledging success, all meteorological variables had negligible effects (Table 1, Table S3). Longer nestling periods (i.e. later ringing of nestlings) were associated with lower fledging success (Table 1). 

**Figure 4:**
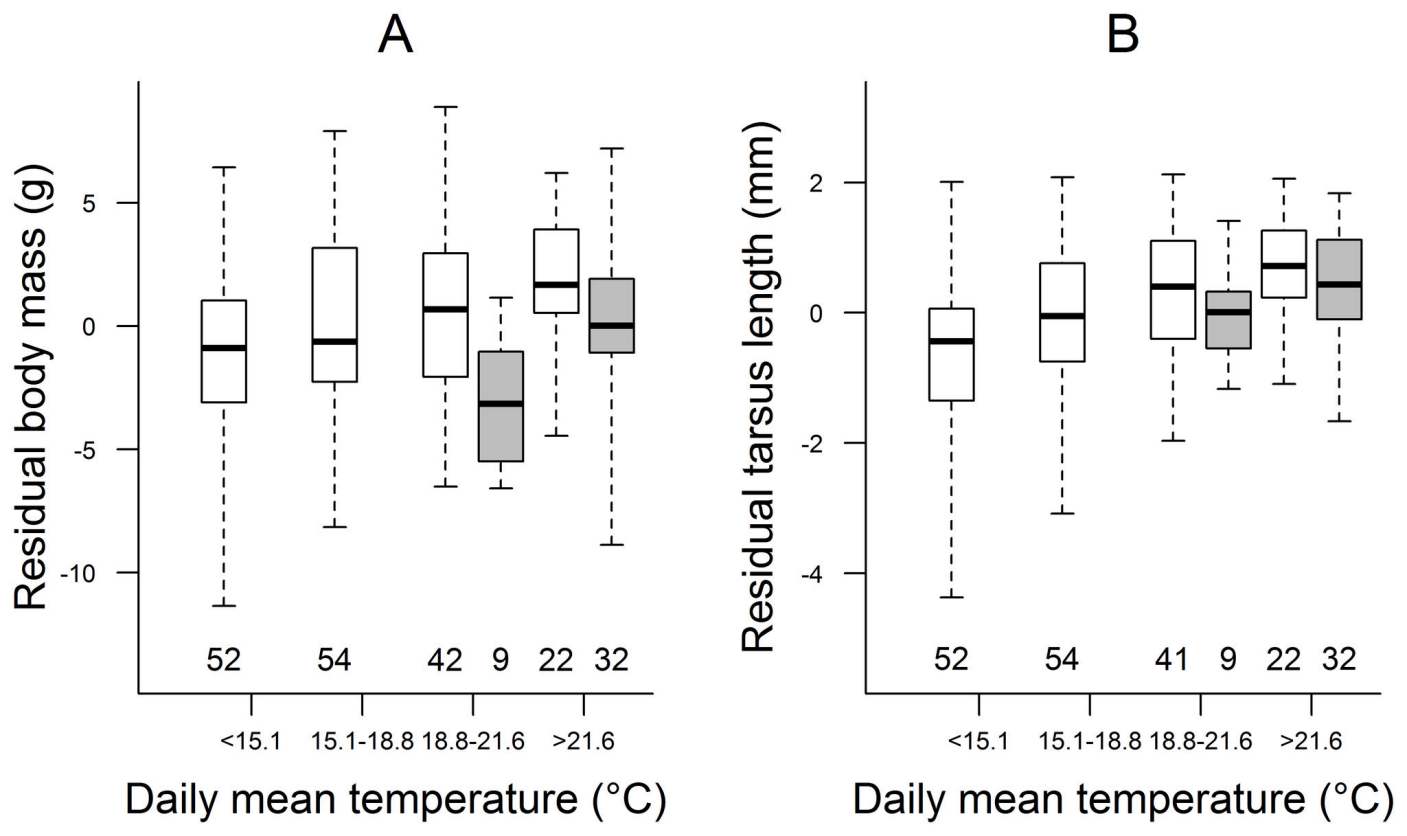
Relationship of nestlings' body size with average daily mean temperature and number of hot days. For illustrative purposes, daily mean temperature was categorized according to its 25%, 50% and 75% percentiles. The number of hot days was dichotomized as few (≤2; white boxes) and many (>2; grey boxes) as the median was zero and the 75% percentile was 2 hot days. Body mass (A) was controlled for date and age of nestings, whereas tarsus length (B) was controlled for brood size and age of nestlings by taking their residuals from linear regressions. Box plots show the median (thick line), interquartile range (box) and the range of data (whiskers); sample sizes (i.e. number of broods) are shown below each box.

**Figure 5:**
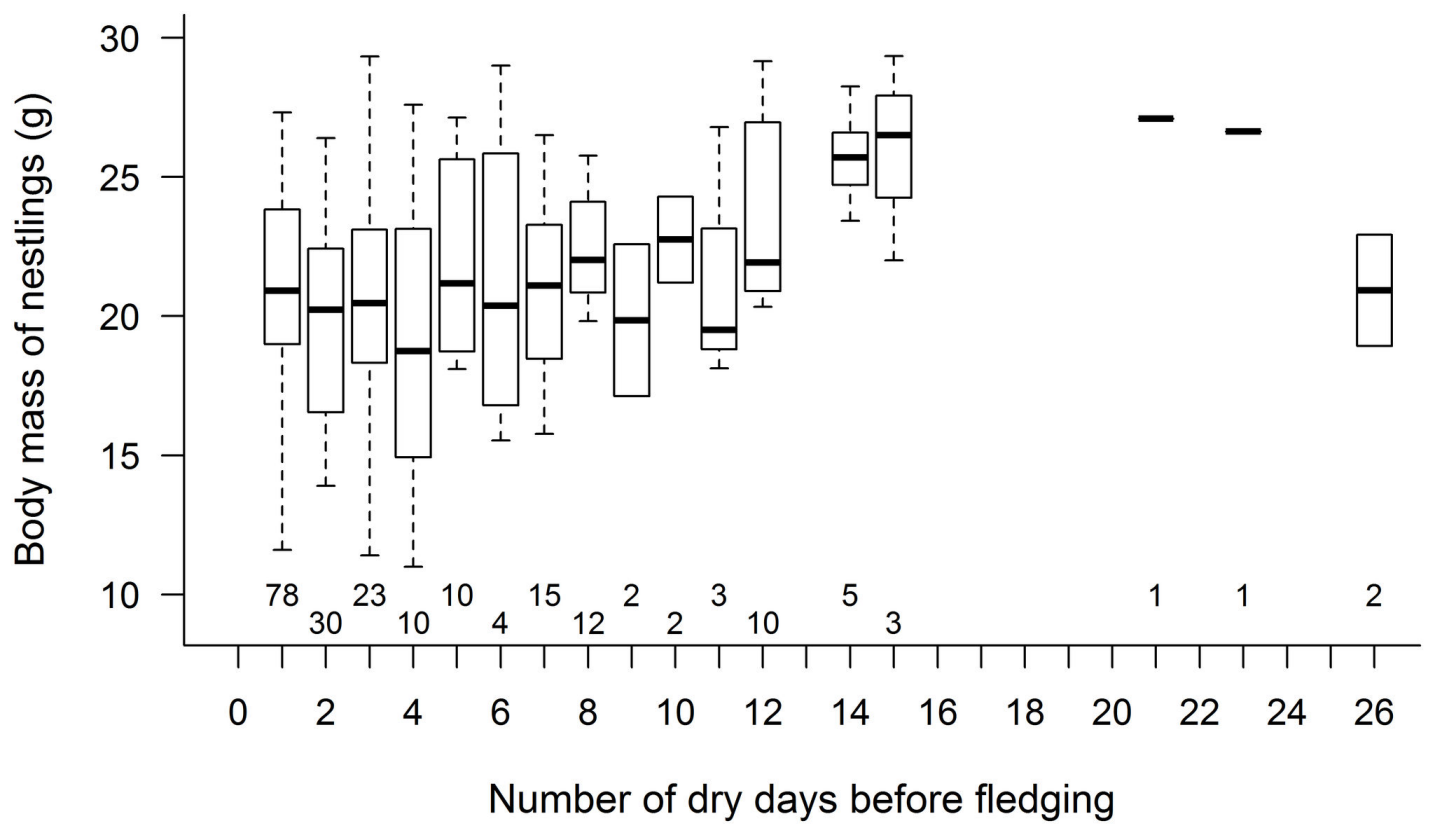
Relationship of nestlings' body mass with the number of dry days before fledging. Box plots show the median (thick line), interquartile range (box) and the range of data (whiskers); sample sizes are shown below each box.

 Both body mass (Figure 4A, Table S4) and tarsus length (Figure 4B, Table S5) of nestlings at pre-fledging age were larger in periods with higher daily mean temperature whereas the frequency of hot days had a smaller opposing effect (Table 1). Furthermore, nestlings weighed more when there was a longer period without rain before fledging (Figure 5, Table 1). Additionally, nestlings that hatched later in the breeding season weighed less, those in bigger broods had longer tarsi, and older nestlings had larger body mass and tarsus length (Table 1). 

 Sex ratio of the 285 nestlings with known sex did not differ significantly from unity when all years were combined (binomial test: 140 males, 49.1%, p = 0.813), and did not differ between years (χ^2^ test: χ^2^_2_ = 1.09, p = 0.581; Table S6). Similarly, the primary sex ratio of 20 clutches (where all the laid eggs hatched) did not differ significantly from unity (40 males out of 85 nestlings, 47%; p = 0.665). Brood sex ratio did not show considerable relationship with any of the studied meteorological variables (Table S6, Table 1). Multigroup analyses showed that models assuming sex-dependent weather effects on nestlings' body size did not fit the data better than models with sex-independent parameter estimates either for body mass (Ĉ_min_=6.63, p=0.250) or tarsus length (Ĉ_min_=0.59, p=0.964).

## Discussion

Our study has revealed several correlations between weather conditions and various components of reproductive success of house sparrows. Our results suggest that warm weather was generally favourable both during the incubation and nestling periods, but extreme heat had a negative effect on nestlings' body size, whereas dry periods up to 2-3 weeks resulted in higher nestling weight. The effect sizes of these relationships were small, but in some cases comparable to the effects of other important determinants of reproductive success such as date and brood size. Furthermore, the 95% confidence intervals of the path coefficients indicated that temperature can have strong effect on nestlings' size. Interestingly, our results indicate that extreme temperatures (i.e. those occurring only in 10% of time in our temperate region) can have differential effects on different aspects of avian reproduction, and these effects can oppose the general effect of average daily temperatures.

 Hatching success increased with the number of hot days, probably because >30°C air temperatures help to maintain the optimal temperature of eggs. The average incubation temperature of house sparrows is 34.2 °C [36]. When parents are not incubating, the temperature of eggs may decrease less if the weather is warm, leading to lower variability in egg temperature and thereby better embryo development. Similarly, hatching success decreased with the number of cold days, but only when the incubation period was relatively short. This interaction probably arose because both hatching success and the length of the incubation period may be related not only to weather but also other variables such as the body condition, age and experience of parents [54,55]. Thus, clutches with incubation periods prolonged due to cold days may be more successful than those prolonged due to poor parental quality, leading to a spurious positive relationship between the number of cold days and hatching success for long incubation periods.

 Fledging success was not related to weather conditions which is surprising as weather extremities can affect nestling mortality directly and also indirectly through food availability in other species [15,16]. A study of British house sparrows [56] found a quadratic relationship between the annual number of independent young raised per pair and both temperature and precipitation within a similar range as our meteorological data. However, since the house sparrow is a multi-brooded species, annual reproductive output is affected by the number of broods raised per year which in turn may also be influenced by weather [46,57], a phenomenon we could not study because not all parents were ringed in our population. Over our 6 study years, length of the reproductive season (from the laying the first egg until the ringing of the last nestling in the colony) tended to increase with yearly mean temperature (Pearson correlation: r=0.77, p=0.075), which suggests at least the possibility of a similar temperature effect in our population as those found in Britain. 

 The correlations we found indicate that, at least within a single reproductive attempt, weather variability in our region has little effect on the proportion of young that reaches the age of fledging, but it can strongly affect the quality of offspring, as both body mass and tarsus length of nestlings were greater under warm weather conditions (i.e. higher mean daily temperature). Larger fledglings have better survival [58–60], so the size and body condition of offspring is an important component of parents’ fitness. Warm weather may promote nestling growth by several mechanisms. First, house sparrow nestlings are poikilothermic for the first ca. 10 days of their life and need brooding from parents to maintain their body temperatures in the thermoneutral zone of 35-40°C [36]. Higher air temperatures may reduce the heat loss of unattended broods and thereby may allow both nestlings and parents to invest less into thermoregulation and more into growth and foraging. Second, nestling development is dependent on provisioning by the parents. In house sparrows, investment by both parents is required to maximize reproductive success [48], and unfavourable weather conditions reduce the provisioning rate of male parents [28]. Nestlings of this species require a diet of >80% arthropod prey [36,61], and weather may affect the activity and abundance of many arthropod taxa resulting in lower success of finding food for nestlings in cold, wet and windy weather [15,62–64].

 Beside the positive effects of generally warm weather, we found that the frequency of extremely hot days was negatively related to nestlings' body size. This is probably due to heat stress, as increasing temperature triggers higher metabolic rate [36] and extreme heat can cause heat shock [65,66]. It seems unlikely that extreme hot temperature reduces the availability of nestlings' food since insects are generally active in warm weathers [26,67]. However, we need further information on parental behaviour, since if parental activities on extremely hot days (e.g. hunting for chicks' food) have significant physiological costs then parents may reduce provisioning even at the expense of reduced growth or survival of current broods. 

 Precipitation had negligible effect on all aspects of reproduction in our study except for the body mass of nestlings: the longer the period of uninterrupted dry weather before fledging, the larger the fledglings’ weight. A possible explanation for this relationship is that insects may be hidden and immobile in rain, thus dry weather may increase food availability and thereby the body condition of nestlings at ringing. 

 Although adult sex ratio is usually slightly male-biased in house sparrow populations [36], in our study the sex ratio of neither hatchlings nor fledglings differ significantly from unity. In parallel with our result that nestling mortality (i.e. fledging success) was unrelated to meteorological variables, we found that offspring sex ratio did not vary with weather conditions. In line with this, in a North-American population of house sparrows, Westneat et al. [68] found little evidence that offspring sex ratio is shifted under good conditions, measured by date and parental characteristics. Furthermore, our results indicated that the effects of weather on nestlings' body size was similar in male and female nestlings. Altogether, these findings do not support that the two sexes differ considerably in environmental sensitivity during early ontogeny and/or in parents’ investment into offspring in this species.

Although correlative studies cannot prove causation, our study highlights the importance of the deeper understanding of weather effects on avian reproduction. For example, the effects of meteorological conditions may be complicated by variation in spring phenology. Although breeding date *per se* had little effect on reproduction in our analyses except that fledglings’ body mass decreased over the season (Table 1), the diferent timings of birds’ egg laying and arthropods’ emergence (i.e. a mismatch in phenology) can be an important determinant of fitness [13,69]. Mismatches can potentially confound the effects of weather, e.g. because warm temperatures might promote nestling growth via food availability in well-timed breeders but not (or less so) in mismatched pairs or years. Further studies are needed to tease apart these effects.

Taken together, we found complex relationships between weather and the reproductive success of house sparrows. Our results indicate that the overall meteorological conditions and extremities can have opposing effects which can vary between different components of fitness. This implies that the consequences of globally rising temperatures and increasing frequency of extreme meteorological conditions are not easy to predict, and detailed studies at the population level are necessary for a better understanding of the impact of weather and climate on population dynamics. 

## Supporting Information

Figure S1 **Seasonal change of daily mean temperature (**A**) and daily amount of precipitation (**B**) in the study area during house sparrow reproduction.** Linear regression line (dashed) and quadratic regression curve (solid) is fitted.
(TIF)

Table S1 **Annual values of reproductive and meteorological parameters in the study population between April-August.**
(DOC)

Table S2 **Model set for hatching success; models with ∆AIC>2 are written in bold (n=262 nests).**
(DOC)

Table S3 **Model set for fledging success; models with ∆AIC>2 are written in bold (n=211 nests).**
(DOC)

Table S4 **Model set for body mass of nestlings; models with ∆AIC>2 are written in bold (n=211 nests).**
(DOC)

Table S5 **Model set for tarsus length of nestlings; models with ∆AIC>2 are written in bold (n= 210 nests).**
(DOC)

Table S6 **Model set for sex ratio at fledging; models with ∆AIC>2 are written in bold (n=61 nests).**
(DOC)
