# Supplementary figures and images for: Effects of Extreme Weather on Reproductive Success in a Temperate-Breeding Songbird
Source: PLoS One. 2013 Nov 5;8(11):e80033. doi: 10.1371/journal.pone.0080033 (PMC3818280; doi:10.1371/journal.pone.0080033)

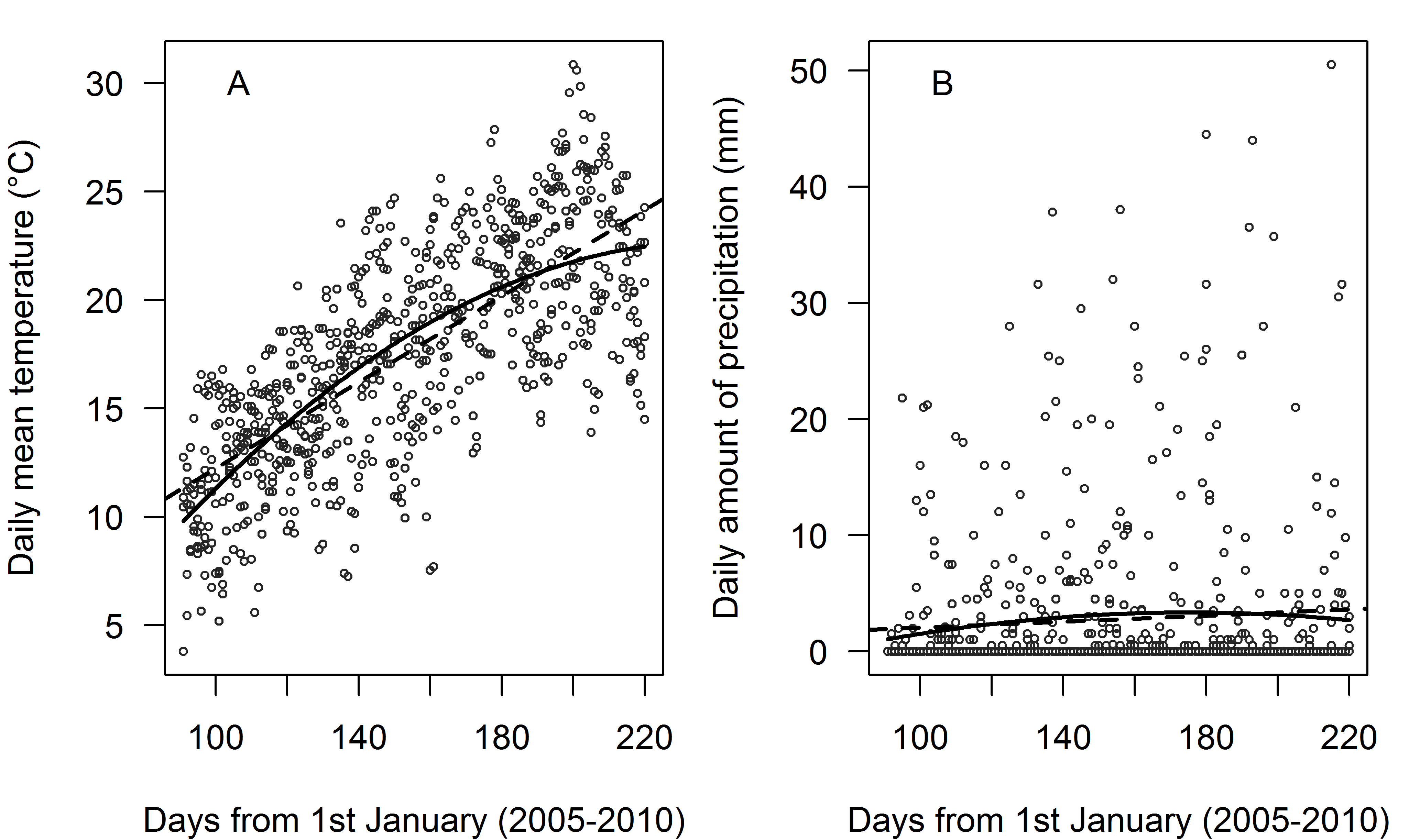

Supplement: Figure S1 — Seasonal change of daily mean temperature (A) and daily amount of precipitation (B) in the study area during house sparrow reproduction. Linear regression line (dashed) and quadratic regression curve (solid) is fitted. (TIF) [file pone.0080033.s001.tif]
