# Supplementary material for: Effects of Extreme Weather on Reproductive Success in a Temperate-Breeding Songbird
Source: PLoS One. 2013 Nov 5;8(11):e80033. doi: 10.1371/journal.pone.0080033 (PMC3818280; doi:10.1371/journal.pone.0080033)
Supplement: Table S1 — Annual values of reproductive and meteorological parameters in the study population between April-August. (DOC) [file pone.0080033.s002.doc]

Table S1: Annual values of reproductive and meteorological parameters in the study population between April-August

|  | **2005** | **2006** | **2007** | **2008** | **2009** | **2010** | **Total** |
| --- | --- | --- | --- | --- | --- | --- | --- |
| total number of eggs | 248 | 296 | 219 | 277 | 265 | 122 | 1427 |
| total number of hatched nestlings | 202 | 227 | 157 | 187 | 189 | 103 | 1065 |
| total number of fledged nestlings | 138 | 152 | 129 | 125 | 134 | 58 | 736 |
| hatchlings (nr.) / eggs (nr.) | 0.815 | 0.767 | 0.717 | 0.675 | 0.713 | 0.844 | 0.746 |
| fledglings (nr.) / hatchlings (nr.) | 0.683 | 0.670 | 0.822 | 0.668 | 0.709 | 0.563 | 0.691 |
| fledglings (nr.) / eggs (nr.) | 0.556 | 0.514 | 0.589 | 0.451 | 0.506 | 0.475 | 0.516 |
| total number of clutches | 62 | 69 | 46 | 59 | 56 | 25 | 317 |
| total number of hatched clutches | 52 | 60 | 40 | 50 | 50 | 25 | 277 |
| total number of fledged clutches | 46 | 50 | 38 | 40 | 40 | 17 | 231 |
| hatched / total clutches | 0.839 | 0.870 | 0.870 | 0.847 | 0.893 | 1.000 | 0.874 |
| fledged / hatched clutches | 0.885 | 0.833 | 0.950 | 0.800 | 0.800 | 0.680 | 0.834 |
| fledged / total clutches | 0.742 | 0.725 | 0.826 | 0.678 | 0.714 | 0.680 | 0.729 |
| mean body mass (g) | 23.53 | 20.84 | 21.92 | 19.26 | 20.76 | 19.56 | 21.14 |
| mean tarsus length (mm) | 18.48 | 17.53 | 17.51 | 16.83 | 17.04 | 17.01 | 17.4 |
| total number of known sexes | 89 | 97 | 99 | - | - | - | 285 |
| total number of males | 40 | 48 | 52 | - | - | - | 140 |
| total number of females | 49 | 49 | 47 | - | - | - | 145 |
| sex ratio (males/total) | 0.449 | 0.495 | 0.525 | - | - | - | 0.491 |
| cluthes with known sex ratio | 21 | 21 | 25 | - | - | - | 67 |
| mean daily average termperature (°C) | 16.55 | 18.02 | 19.18 | 16.37 | 18.82 | 17.81 | 17.87 |
| total number of hot days | 21 | 66 | 44 | 24 | 14 | 21 | 190 |
| total number of cold days | 103 | 76 | 10 | 54 | 42 | 40 | 325 |
| total number of dry days | 168 | 227 | 325 | 195 | 174 | 76 | 1165 |
| total precipitation (mm) | 667.4 | 409.5 | 376.0 | 244.5 | 290.0 | 524.7 | 2512.1 |
| total number of rainy days | 197 | 276 | 110 | 198 | 196 | 115 | 1092 |
| total number of heavy rain days | 48 | 66 | 16 | 66 | 27 | 21 | 244 |
