# Supplementary material for: Effects of Extreme Weather on Reproductive Success in a Temperate-Breeding Songbird
Source: PLoS One. 2013 Nov 5;8(11):e80033. doi: 10.1371/journal.pone.0080033 (PMC3818280; doi:10.1371/journal.pone.0080033)
Supplement: Table S2 — Model set for hatching success; models with ∆AIC>2 are written in bold (n=262 nests). (DOC) [file pone.0080033.s003.doc]

**Table S2: Model set for hatching success; models with ∆AIC>2 are written in bold (n=262 nests**)

| Variables in the models | AIC value | ∆ AIC | Akaike weight |
| --- | --- | --- | --- |
| **Date, I, T, P, H, C, CxI** | **1342.93** | **0.00** | **0.21** |
| **Date, I, T, P, H, C** | **1343.46** | **0.53** | **0.16** |
| **Date, I, T, P, H, C, R, CxI** | **1344.15** | **1.22** | **0.11** |
| **Date, I, T, P, H, C, R** | **1344.45** | **1.51** | **0.10** |
| **Date, I, T, P, H, C, D, CxI** | **1344.87** | **1.93** | **0.08** |
| Date, I, T, P, H, C, D | 1345.28 | 2.35 | 0.06 |
| Date, I, T, P, H, C, D, R, CxI | 1346.13 | 3.20 | 0.04 |
| Date, I, T, P, H, C, D, R | 1346.36 | 3.43 | 0.04 |
| Date, I, T, P, H | 1346.66 | 3.73 | 0.03 |
| Date, I, T, P, C | 1347.22 | 4.28 | 0.02 |
| Date, I, T, P, C, CxI | 1347.30 | 4.37 | 0.02 |
| Date, I, T, P, C, R | 1348.09 | 5.16 | 0.02 |
| Date, I, T, P, H, R | 1348.25 | 5.32 | 0.01 |
| Date, I, T, P, C, R, CxI | 1348.39 | 5.46 | 0.01 |
| Date, I, T, P, H,D | 1348.61 | 5.68 | 0.01 |
| Date, I, T, P, C, D | 1348.77 | 5.84 | 0.01 |
| Date, I, T, P, C, D, CxI | 1349.02 | 6.09 | 0.01 |
| Date, I, T, P, C, D, R | 1349.81 | 6.88 | 0.01 |
| Date, I | 1350.08 | 7.15 | 0.01 |
| Date, I, T, P, H, D, R | 1350.14 | 7.20 | 0.01 |
| Date, I, T, P, C, D, R, CxI | 1350.22 | 7.29 | 0.01 |
| Date, I, P | 1351.15 | 8.22 | <0.01 |
| Date, I, T | 1351.91 | 8.98 | <0.01 |
| Date, I, T, P | 1352.55 | 9.62 | <0.01 |
| Date, I, T, P, R | 1354.15 | 11.21 | <0.01 |
| Date, I, T, P, D | 1354.54 | 11.61 | <0.01 |
| Date, I, T, P, D, R | 1356.11 | 13.18 | <0.01 |

Date=Date of hatching of the first chick

T=daily mean temperature

P=Total amount of precipitation

H=Number of hot days

C=Number of cold days

D=Number of dry days

R=Number of heavy rain days

I=Incubation time

CxI=Interaction between number of cold days and incubation time
