# Supplementary material for: Effects of Extreme Weather on Reproductive Success in a Temperate-Breeding Songbird
Source: PLoS One. 2013 Nov 5;8(11):e80033. doi: 10.1371/journal.pone.0080033 (PMC3818280; doi:10.1371/journal.pone.0080033)
Supplement: Table S3 — Model set for fledging success; models with ∆AIC>2 are written in bold (n=211 nests). (DOC) [file pone.0080033.s004.doc]

**Table S3: Model set for fledging success; models with ∆AIC>2 are written in bold (n=211 nests)**

| Variables in the models | AIC value | ∆ AIC | Akaike weight |
| --- | --- | --- | --- |
| **Date, A** | **1286.17** | **0.00** | **0.22** |
| **Date, A, T** | **1287.28** | **1.11** | **0.13** |
| **Date, A, P** | **1287.54** | **1.37** | **0.11** |
| Date, A, T, P, D | 1288.91 | 2.73 | 0.06 |
| Date, A, T, P | 1288.98 | 2.81 | 0.06 |
| Date, A, T, P, D, R | 1289.51 | 3.34 | 0.04 |
| Date, A, T, P, H, D | 1289.62 | 3.45 | 0.04 |
| Date, A, T, P, H | 1289.74 | 3.57 | 0.04 |
| Date, A, T, P, R | 1289.89 | 3.71 | 0.04 |
| Date, A, T, P, H, C, D, | 1290.09 | 3.91 | 0.03 |
| Date, A, T, P, C, D | 1290.10 | 3.93 | 0.03 |
| Date, A, T, P, C, D, R | 1290.17 | 4.00 | 0.03 |
| Date, A, T, P, C | 1290.23 | 4.06 | 0.03 |
| Date, A, T, P, H, C | 1290.29 | 4.12 | 0.03 |
| Date, A, T, P, H, C, D, R | 1290.49 | 4.32 | 0.03 |
| Date, A, T, P, H, D, R | 1290.55 | 4.38 | 0.03 |
| Date, A, T, P, C, R | 1290.69 | 4.52 | 0.02 |
| Date, A, T, P, H, R | 1290.93 | 4.76 | 0.02 |
| Date, A, T, P, H, C, R | 1291.05 | 4.88 | 0.02 |

Date=Date of hatching of the first chick

T=daily mean temperature

P=Total amount of precipitation

H=Number of hot days

C=Number of cold days

D=Number of dry days

R=Number of heavy rain days

A=Age of nestlings at measuring day
