# Supplementary material for: Effects of Extreme Weather on Reproductive Success in a Temperate-Breeding Songbird
Source: PLoS One. 2013 Nov 5;8(11):e80033. doi: 10.1371/journal.pone.0080033 (PMC3818280; doi:10.1371/journal.pone.0080033)
Supplement: Table S4 — Model set for body mass of nestlings; models with ∆AIC>2 are written in bold (n=211 nests). (DOC) [file pone.0080033.s005.doc]

**Table S4: Model set for body mass of nestlings; models with ∆AIC>2 are written in bold (n=211 nests)**

| Variables in the models | AIC value | ∆ AIC | Akaike weight |
| --- | --- | --- | --- |
| **Date, A, B, T, P, H, D** | **1325.26** | **0.00** | **0.31** |
| **Date, A, B, T, P, D** | **1326.15** | **0.89** | **0.20** |
| **Date, A, B, T, P, H, D, R** | **1327.05** | **1.79** | **0.13** |
| **Date, A, B, T, P, H, C, D,** | **1327.25** | **1.99** | **0.11** |
| Date, A, B, T, P, C, D | 1327.82 | 2.56 | 0.09 |
| Date, A, B, T, P, D, R | 1328.10 | 2.84 | 0.07 |
| Date, A, B, T, P, H, C, D, R | 1329.01 | 3.75 | 0.05 |
| Date, A, B, T, P, C, D, R | 1329.70 | 4.44 | 0.03 |
| Date, A, B, T | 1333.44 | 8.18 | 0.01 |
| Date, A, B, T, P, H | 1334.39 | 9.13 | 0.00 |
| Date, A, B, T, P | 1335.43 | 10.17 | 0.00 |
| Date, A, B, T, P, H, R | 1336.36 | 11.10 | 0.00 |
| Date, A, B, T, P, H, C | 1336.38 | 11.12 | 0.00 |
| Date, A, B, T, P, C | 1337.12 | 11.86 | 0.00 |
| Date, A, B, T, P, R | 1337.42 | 12.16 | 0.00 |
| Date, A, B, T, P, H, C, R | 1338.35 | 13.09 | 0.00 |
| Date, A, B, T, P, C, R | 1339.12 | 13.86 | 0.00 |
| Date, A, B | 1346.76 | 21.50 | 0.00 |
| Date, A, B, P | 1347.17 | 21.91 | 0.00 |

Date=Date of hatching of the first chick

T=daily mean temperature

P=Total amount of precipitation

H=Number of hot days

C=Number of cold days

D=Number of dry days

R=Number of heavy rain days

A=Age of nestlings at measuring

B=Brood size
