# Supplementary material for: Effects of Extreme Weather on Reproductive Success in a Temperate-Breeding Songbird
Source: PLoS One. 2013 Nov 5;8(11):e80033. doi: 10.1371/journal.pone.0080033 (PMC3818280; doi:10.1371/journal.pone.0080033)
Supplement: Table S5 — Model set for tarsus length of nestlings; models with ∆AIC>2 are written in bold (n= 210 nests). (DOC) [file pone.0080033.s006.doc]

**Table S5: Model set for tarsus length of nestlings; models with ∆AIC>2 are written in bold (n= 210 nests)**

| Variables in the models | AIC value | ∆ AIC | Akaike weight |
| --- | --- | --- | --- |
| **Date, A, B, T** | **1319.93** | **0.00** | **0.15** |
| **Date, A, B, T, P, H, C, D,** | **1320.43** | **0.50** | **0.12** |
| **Date, A, B, T, P, H, D** | **1320.61** | **0.69** | **0.11** |
| **Date, A, B, T, P, H, C** | **1320.72** | **0.80** | **0.10** |
| **Date, A, B, T, P, H** | **1320.95** | **1.02** | **0.09** |
| **Date, A, B, T, P, D** | **1321.48** | **1.55** | **0.07** |
| **Date, A, B, T, P** | **1321.91** | **1.98** | **0.06** |
| Date, A, B, T, P, H, D, R | 1322.20 | 2.27 | 0.05 |
| Date, A, B, T, P, H, C, D, R | 1322.28 | 2.35 | 0.05 |
| Date, A, B, T, P, C, D | 1322.58 | 2.65 | 0.04 |
| Date, A, B, T, P, H, C, R | 1322.67 | 2.74 | 0.04 |
| Date, A, B, T, P, H, R | 1322.71 | 2.78 | 0.04 |
| Date, A, B, T, P, C | 1322.99 | 3.07 | 0.03 |
| Date, A, B, T, P, D, R | 1323.31 | 3.39 | 0.03 |
| Date, A, B, T, P, R | 1323.85 | 3.92 | 0.02 |
| Date, A, B, T, P, C, D, R | 1324.53 | 4.60 | 0.01 |
| Date, A, B, T, P, C, R | 1324.99 | 5.07 | 0.01 |
| Date, A, B | 1328.05 | 8.13 | 0.00 |
| Date, A, B, P | 1328.91 | 8.98 | 0.00 |

Date=Date of hatching of the first chick

T=daily mean temperature

P=Total amount of precipitation

H=Number of hot days

C=Number of cold days

D=Number of dry days

R=Number of heavy rain days

A=Age of nestlings at measuring

B=Brood size
