# Supplementary material for: Effects of Extreme Weather on Reproductive Success in a Temperate-Breeding Songbird
Source: PLoS One. 2013 Nov 5;8(11):e80033. doi: 10.1371/journal.pone.0080033 (PMC3818280; doi:10.1371/journal.pone.0080033)
Supplement: Table S6 — Model set for sex ratio at fledging; models with ∆AIC>2 are written in bold (n=61 nests). (DOC) [file pone.0080033.s007.doc]

**Table S6: Model set for sex ratio at fledging; models with ∆AIC>2 are written in bold (n=61 nests)**

| Variables in the models | AIC value | ∆ AIC | Akaike weight |
| --- | --- | --- | --- |
| **Date** | **424.309** | **0** | **0.26** |
| **Date, T** | **426.133** | **1.824** | **0.10** |
| **Date, P** | **426.303** | **1.994** | **0.09** |
| Date, T, P, D | 426.883 | 2.574 | 0.07 |
| Date, T, P, H, D | 427.353 | 3.044 | 0.06 |
| Date, T, P, D, R | 427.637 | 3.328 | 0.05 |
| Date, T, P, R | 427.815 | 3.506 | 0.05 |
| Date, T, P, H, D, R | 427.988 | 3.679 | 0.04 |
| Date, T, P | 428.079 | 3.77 | 0.04 |
| Date, T, P, H, R | 428.136 | 3.827 | 0.04 |
| Date, T, P, H | 428.555 | 4.246 | 0.03 |
| Date, T, P, C, D | 428.655 | 4.346 | 0.03 |
| Date, T, P, H, C, D, | 428.725 | 4.416 | 0.03 |
| Date, T, P, H, C, R | 429.222 | 4.913 | 0.02 |
| Date, T, P, H, C, D, R | 429.326 | 5.017 | 0.02 |
| Date, T, P, C, D, R | 429.4 | 5.091 | 0.02 |
| Date, T, P, C, R | 429.425 | 5.116 | 0.02 |
| Date, T, P, H, C | 429.626 | 5.317 | 0.02 |
| Date, T, P, C | 429.661 | 5.352 | 0.02 |

Date=Date of hatching of the first chick

T=daily mean temperature

P=Total amount of precipitation

H=Number of hot days

C=Number of cold days

D=Number of dry days

R=Number of heavy rain days
